# Brain death diagnosis and apnea test safety

**DOI:** 10.4103/0972-2327.56326

**Published:** 2009

**Authors:** Calixto Machado, Jesus Perez, Claudio Scherle, Alejandro Areu, Alejandro Pando

**Affiliations:** Institute of Neurology and Neurosurgery, Department of Clinical Neurophysiology, Havana, Cuba; 1 Hermanos Ameijeiras Hospital, Service of Neurology, Havana, Cuba

**Keywords:** Ancillary tests, apnea test, brain death

## Abstract

The apnea test is a mandatory examination for determining brain death (BD), because it provides an essential sign of definitive loss of brainstem function. However, several authors have expressed their concern about the safety of this procedure as there are potential complications such as severe hypotension, pneumothorax, excessive hypercarbia, hypoxia, acidosis, and cardiac arrhythmia or asystole. These complications may constrain the examiner to abort the test, thereby compromising BD diagnosis. Nevertheless, when an appropriate oxygen-diffusion procedure is used, this technique is safe. We review here the prerequisites to begin the test, its procedure, potential complications, and the use of alternative ancillary tests. We recommend that the apnea test be retained as a mandatory procedure for the diagnosis of BD. In those situations when the apnea test is terminated by the examiner for some reason or when it is impossible to carry it out in a patient due to the presence of some pathologic condition, alternative ancillary tests should be used to confirm BD.

## Introduction

The apnea test (AT) has been considered by most authors as the ‘condition *sine qua non*’ for determining brain death (BD) because it provides an essential sign of a definitive loss of brainstem function.[1–7] Nonetheless, it is the most difficult clinical test in BD protocols and, besides, is potentially harmful and lengthy. The possible complications of this test include severe hypotension, pneumothorax, excessive hypercarbia, hypoxia, acidosis, and cardiac arrhythmia or asystole. The occurrence of any of these complications may constrain the examiner to abort the test, thereby compromising BD diagnosis.[1–7] However, when an appropriate oxygen-diffusion procedure is used, this technique is safe.[8–16] We review here the prerequisites for conducting the test, the procedure, the potential complications, and the use of alternative ancillary tests.

## Prerequisites for the apnea test

It is important to ensure that certain prerequisites are satisfied before the AT is carried out.[14,15] These are as follows:

The body temperature of the subject needs to be 32°C or more. Most authors are of the opinion that this test should not be begun when the body temperature is below 32°C. It may be necessary to warm the body to 36°C if the body temperature is low. Correction of hypothermia facilitates CO_2_ production and reduces the chances of hypotension by stabilizing blood pressure and the hemodynamic state.[17–20]

The required PaO_2_ levels are often not clearly defined; however, it is recommended that preoxygenation with 100% O_2_ be done for some time, generally for 10 min, and that hypoxia be avoided. Some experts recommend maintenance of a normal PO_2_ or preoxygenation to obtain an arterial PO_2_ ≥ 200 mmHg.[12,19] Preoxygenation helps to avoid possible hypocapnia, which may be due to hyperventilation, or to the setting of high tidal volumes on the mechanical ventilator, or if the patient if hypothermic.

It is important to ensure that the arterial PCO_2_ or PaCO_2_ is normal or above 40 mmHg.^23^ Certain authors recom_m_ end a starting PaCO_2_ of 36 mmHg or higher.[21–22] Others state that the important consideration is to elevate the PaCO2 to 20 mmHg above the starting PaCO2 level.[23]

It is recommended that the blood pH should be normal or in the low basic range before beginning the AT.[24]

A pretest systolic blood pressure of at least 90 mmHg is recommended by the Quality Standards Subcommittee of the American Academy of Neurology.

Fluid balance: Euvolemia or a positive fluid balance during the previous 6 h[15,19] is also recommended.

Medication: AT should not be performed when the subject is under the influence of drugs that may paralyze the respiratory muscles, i.e., relaxants such as pancuronium.[25]

## Procedure of the apnea test

### Disconnection of the patient from the respirator

If continuous or intermittent oxygen supply is preceded by denitrogenation of blood gases, high PaO_2_ levels can be sustained for very long periods of time.[26] Preoxygenation removes alveolar nitrogen stores and facilitates oxygen transport.[27] There are several techniques for ascertaining that there is sufficient oxygenation during AT.[15] The first method is to disconnect the patient from the respirator and to insert a catheter or cannula into the endotracheal tube down to the level of the carina and provide pure oxygen at a rate of 4–10 l/min. This would ensure sufficient alveolar ventilation and transport of oxygen to the blood even without any respiratory movements.[17]

In the second procedure the patient is not disconnected from the respirator but the minute volume is decreased to a very low level (0.5–2 l/min), with the respirator in the synchronized intermittent mandatory volume ventilation mode and with pure oxygen provided for inspiration. In this procedure, the patient is not disconnected until the required PaCO_2_ is achieved. Lang and coworkers prefer this method as it prevents tracheopulmonary complications and allows the examiner to detect any spontaneous respiratory effort.[14,15] Al Jumah *et al*, have proposed a third procedure of biphasic intermittent positive airway pressure (BIPAP), a method known as ‘bulk diffusion’.[28]

Some authors considered it safer to test for apnea by keeping the patient with a continuous flow of 100 % oxygen and low positive end expiratory pressure (PEEP), than to disconnect them from the ventilator.[29,30]

### Duration of the apnea test

Apnea is concluded when no breathing effort is observed at a PaCO_2_ of 60 mm Hg or with a 20 mm Hg increment from baseline; this indicates that the AT is positive, thereby supporting the diagnosis of BD. Since a rapid increase in PaCO_2_ to 20 mmHg above normal baseline is believed to provide a maximal stimulus to the respiratory centers, such an increase is recommended when the baseline PaCO_2_ is at or above 36–40 mmHg. On the other hand, if respiratory movements are detected, the AT is classified as negative (i.e., not supportive of a BD diagnosis), the patient should be reconnected to the respirator. If arterial pressure drops to < 90 mm Hg, or a noticeable desaturation is detected by the oximeter or cardiac arrhythmias occur, the procedure should be stopped immediately and the ventilator should be connected. Some authors do not consider the duration of the AT to be relevant,[15] while others have recommended that the test be stopped after 10–15 min, even if blood gas levels cannot be determined.[25,31] Under physiological states, the PaCO_2_ is predicted to increase by 3-4 mmHg in such situations[32] but this may vary considerably under conditions of brain death. According to some authors it is essential to monitor the blood gases at frequent intervals and the procedure can be extended up to 1 h.[15]

An alternative procedure for the AT has been proposed when a decrease in ventilator volume is not desirable. In this procedure, CO_2_ is insufflated to achieve the desired PaCO_2_ levels. Lang *et al*. considered this method to be particularly useful in cases where there is minimal rise in PaCO_2_ or when blood oxygenation is hampered by pulmonary problems.[33–35] This technique requires close monitoring of the patient, but the duration of the procedure could be shortened considerably.[14,15]

Several authors have estimated the level of PCO_2_ at which maximal stimulation of the medullary respiratory centers would occur. Wijdicks has reported the occurrence of a probable unsafe hypercarbia in some cases who had started to breathe at low PCO_2_ levels (about 30–35 mm of Hg) but lost respiratory drive some hours later.[10,36] The same group has also reported that when the ventilator settings are below certain trigger sensitivity thresholds, some patients show false readings of spontaneous respiratory movements at rates of 20–30/min, which may cause confusion in BD determination.[37]

### Complications

Several complications can occur during the AT. Goudreau *et al*, have reported that approximately one in four ATs were associated with cardiovascular complications, and the rate of complications nearly doubled when the tests were carried out without satisfying the prerequisites. Hypotension was the most frequent complication (24%), while cardiac arrhythmia with the potential for ventricular fibrillation or arrest was much less common (1%). A blood pressure reading of 90 mmHg before testing is acceptable as a rule. Usually there is mild increase in blood pressure with hyperoxygenation and marked decrease with hypercapnia. Persistent hypotension may be corrected using intravenous fluids, 5% albumin, or an increase of intravenous dopamine or (nor)epinephrine infusion.[10] It is considered that hypoxemia contributes to cardiac arrhythmia and hypotension during AT.[25] If pulse oximetry is used, saturation values should not drop below 80% as hypoxemia can lead to cardiac arrhythmia or hypotension during AT.[25] There are no precise standards for the blood pH, although pH values less than 7.2 or 7.0 should be avoided although there are reports of safe conduct of the test in spite of very low blood pH values.[38,39] Low pH of blood is highly correlated with raised PaCO_2_ and it is rapidly restored with normoventilation or mild hyperventilation. Normalization of blood pH by administration of buffer or alkaline solutions should be avoided as it could lead to alkalosis when normal ventilation is resumed.

Saposnik *et al*, reported complications in more than two-thirds of their patients. These complications included hypotension (12%), acidosis (68%), and hypoxemia (23%). They reported that four patients suffered major complications (e.g., pneumothorax).[40] Barotrauma is another complication that has been reported following AT.[41–44]

Coimbra directly critiqued the use of AT, considering that it may induce potentially irreversible collapse of intracranial vessels by simultaneously worsening intracranial hypertension and inducing hypotension in brain-dead patients. This author remarked that “apnea testing may induce rather than diagnose irreversible brain damage”.[40] This argument has been rejected by other authors, because “It has never been demonstrated that global cerebral ischemic penumbra is transformed into global brain infarction by AT,” suggesting future studies using serial PET assessments.[24]

### Is the apnea test safe in BD diagnosis?

Most authors are of the opinion that in spite of the potential complications, the AT is safe when performed after appropriate oxygen-diffusion procedures.[8–16] In the rare situations where AT cannot be carried out, other ancillary tests may be required.[14,15,33–35,45]

In a large series of 228 BD determinations, with a special emphasis on the AT, it was observed that in 7% instances the physician decided not to administer the AT because of hemodynamic instability or poor oxygenation at baseline, and in 3% cases the test had to be terminated due to hypotension or hypoxemia.[46] In a previous study, hypotension occurred in 24% of ATs. It is not clear if there was an overlap of these two series, because the first study was carried out from 1990 to 1999[10] and the second one from 1996 to 2007.[46]

### Alternative ancillary tests

The AT test may have to be abandoned if the patient has significant hemodynamic instability, poor PaO_2_ at baseline despite adequate preoxygenation, or inability to achieve target PaCO_2_ levels. Extensive thoracic trauma or significant pulmonary disorders interfering with ventilation are other conditions that may prevent the use of the AT.[1–7] Estimation of cerebral blood flow by transcranial Doppler studies or CT angiography can be considered when the AT cannot be carried out. Other ancillary tests that may be used to confirm brain death include multimodality evoked potential studies and the atropine test.[47–56]

## Conclusion

With this study conducted in Cuba, we have arrived at the same conclusion as other authors, i.e., that in all cases in which the AT had to be aborted due to progressive hypotension or hypoxemia, there had been a nonsatisfactory preoxygenation technique.[8–16]

As this test provides an essential sign of a definitive loss of brainstem functions, we recommend that it be retained as a mandatory test in the diagnostic criteria for BD. In those situations when it is aborted by the examiner for some reason or when it is impossible to apply the test in a patient due to certain pathologic conditions, alternative ancillary tests should be used to confirm BD.[47–56]
